# Role of Prokineticin‐2 and Cytokines in Synovial Fluid in Traumatic Anterior Cruciate Ligament Injuries

**DOI:** 10.1155/mi/6435678

**Published:** 2026-09-09

**Authors:** Marco Crippa, Silvia Franchi, Laura Rizzi, Paola Sacerdote, Giulia Galimberti, Laura Antolini, Massimiliano Piatti, Nicola Portinaro, Giada Amodeo, Giovanni Zatti, Antonio Torsello, Marco Turati, Marco Bigoni

**Affiliations:** ^1^ Orthopedic Department, Fondazione IRCCS San Gerardo dei Tintori, 20900, Monza, Italy; ^2^ School of Medicine and Surgery, University of Milano-Bicocca, 20900, Monza, Italy, unimib.it; ^3^ Transalpine Center of Pediatric Sports Medicine and Surgery, University of Milano-Bicocca, 20900, Monza, Italy, unimib.it; ^4^ Department of Pharmacological and Biomolecular Sciences, University of Milan, 20129, Milan, Italy, unimi.it; ^5^ Bicocca Center of Bioinformatics, Biostatistics and Bioimaging (B4 Centre), School of Medicine and Surgery, University of Milano-Bicocca, 20900, Monza, Italy, unimib.it; ^6^ Department of Orthopedic Surgery, Policlinico San Pietro, 24036, Ponte San Pietro, Italy; ^7^ Department of Pediatric Surgery, Fondazione IRCCS Ca’ Granda, Ospedale Maggiore Policlinico, 20122, Milan, Italy, policlinico.mi.it

**Keywords:** anterior cruciate ligament tear, cytokines, meniscal injury, post-traumatic osteoarthritis, prokineticin-2, synovial fluid

## Abstract

**Background:**

Anterior cruciate ligament (ACL) tears induce a proinflammatory intra‐articular environment. Despite successful ACL reconstruction (ACLR), post‐traumatic osteoarthritis (PTOA) incidence remains high. The role of prokineticin‐2 (PK2), a novel inflammatory and pain mediator, was investigated in this observational study of traumatic ACL injuries, associated with meniscal tears.

**Methods:**

Twenty patients with isolated ACL tears (ACL group) and 20 with associated meniscal injury (ACL + M group) were enrolled. Synovial fluid PK2, IL‐10, and TNF‐α levels were measured using ELISA method. Patients underwent long‐term follow‐up (minimum 5 years) with clinical reassessment using patient‐reported outcome measures (PROMs) (IKDC, Lysholm, Tegner activity scale, SKV).

**Results:**

PK2 was detected in both ACL and ACL + M groups without significant differences. Higher PK2 levels were associated with hemarthrosis and correlated positively with IL‐10. No associations were found between PK2 levels and demographic factors or time from injury. At follow‐up, PK2 negatively correlated with IKDC, Lysholm, and SKV scores.

**Conclusion:**

This study provides initial evidence of PK2 in synovial fluid following ACL injury, suggesting its potential role in knee inflammation. PK2 may represent a candidate biomarker involved in the post‐traumatic inflammatory microenvironment; however, further longitudinal studies including structural outcomes are needed.

## 1. Introduction

Knee osteoarthritis (OA) is a degenerative joint disease characterized by chronic pain, inflammation, and cartilage degradation [1]. A specific subtype, known as post‐traumatic OA (PTOA), develops following joint injury [2]. Distinguishing PTOA from idiopathic OA is essential for understanding the heterogeneity of these conditions and for identifying injury‐related biological pathways that may inform future therapeutic strategies.

PTOA often affects younger and healthier individuals compared to those with idiopathic OA, making it a critical area of study [3].

Anterior cruciate ligament (ACL) injuries are among the primary risk factors for knee PTOA, with other contributors including meniscal tears, patellar dislocations, and other ligament lesions [4]: the reported prevalence of PTOA following ACL injuries can reach as high as 87% [5].

Adolescents and young adults participating in sports requiring pivoting and directional changes are particularly vulnerable, with young women facing a 3–5 times higher risk than that of men in these activities [5]. Additionally, the number of arthroscopic interventions is increasing, even among young athletes [6].

Key factors influencing PTOA risk include age, gender, body mass index (BMI), smoking, physical activity levels, and the time elapsed between injury and surgical intervention [7].

Although ACL reconstruction (ACLR) is widely performed to restore joint stability and function, it does not consistently prevent the development of PTOA [8–10]. Both persistent intra‐articular inflammation and altered knee biomechanics after ACL injury and reconstruction have been associated with cartilage degeneration and may contribute to the progression of post‐traumatic joint disease [11, 12].

Prokineticin‐2 (PK2) is a novel inflammatory mediator, belonging to a chemokine family, implicated in immune regulation, pain signaling, and tissue homeostasis, but its role in ACL injuries and subsequent PTOA remains unclear [13]. Prokineticins exert their physiological functions through G‐protein‐coupled receptors (GPCRs), PKR1 and PKR2, and have been investigated as potential therapeutic targets and diagnostic biomarkers in several pathological conditions, including cardiovascular, metabolic, neural, pain‐related, and oncological diseases [13].

Previous studies from our group have identified the presence of PK2 in the synovial fluid of patients with traumatic meniscal injuries, highlighting its potential involvement in intra‐articular inflammatory responses after a traumatic event [14]. Moreover, we recently demonstrated, in a preclinical model of OA, a role of PK2 in the inflammatory and neuroinflammatory pathways involved in OA pathophysiology and pain [15]. The levels of PK2 were upregulated simultaneously to an increase in proinflammatory cytokines and MMP13 levels in the knee joint of OA mice, contributing to local inflammation and pain sensitization.

In the present study, we investigated PK2 levels in the synovial fluid of patients with isolated ACL tears and in patients with associated meniscal injury. Additionally, we determined the synovial fluid levels of TNF‐α, a proinflammatory cytokine involved in cartilage catabolism and joint inflammation in OA, and levels of IL‐10, an anti‐inflammatory cytokine usually modulated following trauma [16]. After at least 5 years post‐arthroscopic surgery, patients underwent comprehensive clinical evaluation: to assess knee function, participants completed four validated patient‐reported outcome measures (PROMs).

By exploring the interplay between PK2, cytokines, and clinical outcomes, this study aims to provide novel preliminary insights into the post‐traumatic inflammatory microenvironment following ACL injury while generating hypotheses for future mechanistic and longitudinal studies.

## 2. Methods

This was an observational cohort study based on prospectively collected synovial fluid samples from patients with traumatic ACL tears, with long‐term clinical follow‐up performed at a minimum of 5 years after arthroscopic treatment.

### 2.1. Patient Population

The study population consisted of 40 patients divided into two groups of 20 patients each: an ACL group with isolated anterior cruciate ligament tears and an ACL + M group with ACL tears and associated traumatic meniscal tears. All patients were recruited from the emergency department, after diagnosis was made by a senior orthopedic surgeon including patient history, physical examination, MRI imaging, and final arthroscopic visualization. Synovial fluid samples were collected either at initial presentation in the emergency department, when a clinically relevant knee effusion was present, or intraoperatively during subsequent arthroscopic surgery. The interval between the traumatic injury and synovial fluid collection was recorded for each patient.

All participants provided written informed consent after receiving detailed information about the study protocol at the time of sample collection. The study was approved by the Ethics Committee and adhered to the WMA Declaration of Helsinki guidelines.

Patients were excluded if they had previous surgical intervention or lesions in the index or contralateral knee, bone or ligament injuries, chondral damage of grade 2 or higher based on the Outerbridge classification [17], arthritis or chronic inflammatory conditions, use of more than three medications, or intra‐articular drug injections.

To assess the influence of time elapsed from traumatic injury on synovial fluid characteristics, patients were categorized into three groups based on the time interval between the injury and fluid collection. An acute group (A) included patients with fluid sampled within 3 days postinjury, a subacute group (SA) comprised those with fluid collected between 3 and 90 days postinjury, and a chronic group (C) included patients with synovial fluid collected more than 90 days after the injury.

### 2.2. Synovial Fluid Collection and Processing

Synovial fluid was obtained via medial or superolateral knee access using a 20‐gauge needle following sterile chlorhexidine preparation. No joint lavage was performed. The aspirate was transferred to sterile 15 mL tubes, immediately placed on ice, and transported to the laboratory. To remove cellular components and debris, samples were centrifuged at 3000 *g*. Supernatants were stored at −80°C until analysis [14].

### 2.3. PK2 and Cytokine Measurement

Synovial fluid cytokines were measured in undiluted samples using specific ELISA kits previously used for synovial fluid samples and shown to provide reproducible data [14, 18]. PK2 levels were quantified using a human PK2 enzyme‐linked immunosorbent assay (ELISA) kit [14] (LS‐F5959, LifeSpan Biosciences, Seattle, WA, USA. Sensitivity: typically less than 246.91 pg/mL; intra‐assay CV <10%; inter‐assay CV <12%) according to the manufacturer’s protocol. TNF‐α and IL‐10 concentrations were determined using specific ELISA kits [14] (DY 210 for TNF‐α and DY217B or high‐sensitive HS100C for IL‐10, R&D Systems, Minneapolis, MN, USA; sensitivity TNF‐α 15.6 pg/mL; IL‐10: 0.03–0.17 pg/mL). All samples were run in duplicate. PK2 and cytokine levels were expressed as pg/mL. To avoid sample deterioration due to freeze–thaw cycles, all biochemical evaluations (ELISA tests) were performed at the same time.

### 2.4. Clinical Follow‐Up

Patients underwent comprehensive clinical evaluations five or more years post‐arthroscopic surgery. To assess knee function, participants completed four PROMs: the Tegner activity scale (range 0–10, quantifying pre‐ and postinjury activity levels) [19], the Lysholm knee score (range 0–100, reflecting eight symptom‐based knee function domains) [20], the International Knee Documentation Committee (IKDC) 2000 subjective knee evaluation form (range 0–100, assessing overall clinical, activity, and functional status) [21], and the simple knee value (range 0–100, capturing patient‐reported knee function) [22].

### 2.5. Statistical Analysis

Continuous variables were summarized using the mean, median, standard deviation, and interquartile range (IQR), while categorical variables were reported as absolute frequencies and percentages. Descriptive statistics were calculated for the overall cohort and stratified by study groups or subgroups.

To compare the distribution of continuous variables between exposure levels, ordinal logistic regression models were applied as an alternative to the Mann–Whitney *U* test, as described by Harrell [23].

In these models, the continuous variable was treated as the outcome and group membership as the regressor. When the outcome variable was binary, the same modeling framework corresponded to standard logistic regression. Furthermore, the ordinal logistic regression models were extended to include additional covariates as regressors. This modeling approach was chosen due to the limited sample size, which made testing the Gaussian assumption unreliable, and to obtain confidence intervals for the exposure effect expressed as an odds ratio (OR).

The relationship between continuous variables was evaluated using the Spearman rank correlation coefficient. Statistical significance was set at *p* < 0.05. To account for multiple comparisons, the Bonferroni correction was applied to the significance threshold. Continuous variable distributions were visually assessed using boxplots. All statistical analyses were performed using Stata 17 and GraphPad Prism 9.

## 3. Results

The ACL group comprised 20 patients (16 males, four females) aged 18–43 years (median age 21 years, IQ‐range 20–31). Patients were classified as either A (*n* = 8), SA (*n* = 8), or C (*n* = 4). Synovial fluid analysis revealed hydrarthrosis in 12 patients and hemarthrosis in eight (Table 1).

**Table 1:** Patient characteristics continuous variables are presented as median and quartiles, while categorical data are expressed as absolute frequencies and percentage values.

Patient characteristics	ACL group (*N* = 20)	ACL+M group (*N* = 20)	Total (*N* = 40)
Gender			
Male	16 (80.0)	12 (60.0)	28 (70.0)
Female	4 (20.0)	8 (40.0)	12 (30.0)
Age (years)	21 (20–31)	24 (19–27)	21 (19–29)
Weight (kg)	72 (64–80)	74 (60–83)	73 (64–83)
Height (cm)	176 (170–183)	176 (174–184)	176 (172–183)
Timing			
Acute (A)	8 (40.0)	3 (15.0)	11 (27.5)
Subacute (SA)	8 (40.0)	13 (65.0)	21 (52.5)
Chronic (C)	4 (20.0)	4 (20.0)	8 (20.0)
Synovial fluid			
Hydrarthrosis	12 (60.0)	12 (60.0)	24 (60.0)
Hemarthrosis	8 (40.0)	8 (40.0)	16 (40.0)
Meniscal tear laterality			
Medial	—	11 (55.0)	—
Lateral	—	9 (45.0)	—

Abbreviations: ACL, anterior cruciate ligament; M, meniscal tear.

The ACL+M group consisted of 12 male and eight female patients aged 18–40 years (median age 24 years, IQ‐range 19–27). Based on time elapsed between injury and fluid collection, patients were subdivided into three A, 13 SA, and four C patients. Eleven patients presented with medial meniscus tears, whereas nine had lateral meniscus tears, 12 hydrarthrosis, and eight hemarthrosis. All patients underwent a standardized arthroscopic partial selective meniscectomy prior to ACLR during the same surgical session.

At the 5‐year follow‐up, patients underwent reassessment using PROMs (Table 2).

**Table 2:** Patient outcomes patient‐reported outcomes (PROMs) are presented as median and quartiles.

PROMs	ACL group (*N* = 11)	ACL + M group (*N* = 19)
Tegner preinjury	6 [5–7]	9 [7–9]
Tegner after injury	6 [5–7]	8 [6–9]
Lysholm	95 [90–100]	91 [83–96]
IKDC	90.1 [83.9–97.7]	86.2 [80.5–95.4]
SKV	90 [80–95]	86 [80–95]

Abbreviations: ACL, anterior cruciate ligament; IKDC, International Knee Documentation Committee subjective knee evaluation form; M, meniscal tear; SKV, simple knee value.

Synovial fluid levels of PK2, TNF‐α, and IL‐10 were determined for all participants and are presented in Table 3 (ACL patients) and Table 4 (ACL + M patients).

**Table 3:** Synovial fluid concentrations of PK2, TNF‐α, and IL‐10 in ACL patients.

ACL group	*n*	PK2 (pg/mL)	IL‐10 (pg/mL)	TNF‐α (pg/mL)
Overall	20	511.6 (419.5–789.5)	3.4 (2.7–10.3)	10.0 (7.2–52.6)
Gender				
Male	16	539.9 (420.6–868.7)	3.1 (2.6–10.3)	10.0 (6.5–63.8)
Female	4	471.9 (334.9–629.6)	3.9 (3.3–7.8)	14.7 (7.3–37.3)
Timing				
Acute (A)	8	706.2 (329.6–996.2)	11.0 (5.9–16.8)	9.3 (6.8–14.2)
Subacute (SA)	8	511.6 (414.7–737.2)	3.1 (2.7–3.9)	20.9 (7.6–58.2)
Chronic (C)	4	467.1 (436.9–560.7)	2.7 (2.3–2.8)	6.2 (6.0–348.6)
Synovial fluid				
Hydrarthrosis	12	467.1 (414.7–539.9)	2.8 (2.3–3.1)	7.9 (6.5–63.8)
Hemarthrosis	8	789.5 (533.7–1035.6)	11.0 (7.9–16.8)	10.9 (8.0–18.3)

*Note:* Data are presented as median and quartiles.

**Table 4:** Synovial fluid concentrations of PK2, TNF‐α, and IL‐10 in ACL + M patients.

ACL + M group	*n*	PK2 (pg/mL)	IL‐10 (pg/mL)	TNF‐α (pg/mL)
Overall	20	532.8 (363.5–680.7)	3.3 (2.5–6.0)	10.5 (7.2–49.3)
Gender				
Male	12	487.6 (363.5–680.4)	3.0 (2.1–3.7)	8.0 (6.6–15.0)
Female	8	572.3 (410.0–715.8)	4.1 (2.9–10.1)	17.8 (11.0–133.5)
Timing				
Acute (A)	3	838.6 (306.7–1253.0)	3.1 (2.6–11.7)	11.8 (8.1–187.9)
Subacute (SA)	13	525.4 (428.0–592.9)	3.6 (2.8–7.3)	10.2 (7.0–19.5)
Chronic (C)	4	442.6 (330.4–565.5)	2.3 (1.7–3.1)	13.6 (7.4–49.2)
Synovial fluid				
Hydrarthrosis	12	519.4 (330.4–572.3)	3.3 (2.2–4.2)	11.3 (8.0–49.2)
Hemarthrosis	8	680.7 (402.0–932.3)	3.5 (2.7–10.3)	7.9 (6.6–64.6)
Laterality				
Medial	11	525.4 (315.7–592.9)	3.1 (2.3–3.8)	11.8 (7.0–19.5)
Lateral	9	553.7 (428.0–768.5)	3.6 (2.8–7.3)	8.4 (7.9–109.7)

*Note:* Data are presented as median and quartiles.

### 3.1. Effect of Injury Type and Patient Characteristics on PK2 and Cytokine Levels

**Figure 1:**
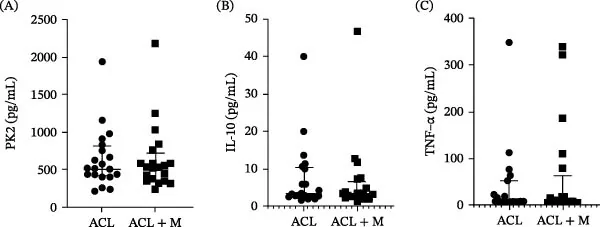
Synovial fluid concentrations of PK2 and cytokines. (A–C) depict PK2, IL‐10, and TNF‐α levels, respectively, in synovial fluid from isolated ACL tear (ACL, *n* = 20) and ACL + traumatic meniscal tear (ACL + M, *n* = 20) patients. Data are plotted as individual points (pg/mL), with the median and interquartile range (IQR,q1–q3) highlighted on the graph. Comparisons between groups were conducted using the ordinal logistic regression model with group as binary covariate.

PK2 was detectable in all synovial fluid samples (Figure 1A). However, no statistically significant differences in PK2 levels were observed between the ACL and ACL + M groups. Ordinal logistic regression did not show a statistically significant difference in PK2 levels between the ACL + M and ACL groups. The odds of exceeding any given PK2 threshold were comparable between groups (OR 0.94, 95% CI: 0.32–2.77; *p* = 0.912).

Similarly, IL‐10 and TNF‐α concentrations did not differ significantly between the two groups (Figure 1B,C). The odds of exceeding any given threshold were comparable between AC + M and ACL patients for both IL‐10 (OR 0.73, 95% CI: 0.25–2.16; *p* = 0.573) and TNF‐α (OR 1.08, 95% CI: 0.36–3.22; *p* = 0.886). Additionally, meniscal laterality did not influence PK2 or cytokine levels in TM patients. No significant correlation was found between patients’ demographic characteristics (gender, weight, and height) and the levels of the three cytokines analyzed in both groups (data not shown).

**Figure 2:**
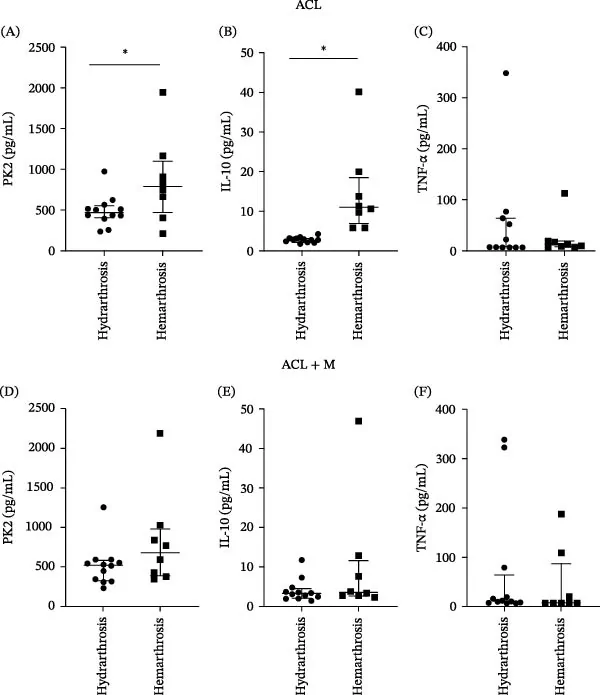
Impact of synovial fluid appearance. PK2 (A and D), IL‐10 (B and E), and TNF‐α (C and F) levels (pg/mL) were compared between hemarthrosis and hydrarthrosis samples from the isolated ACL tear (ACL: hydrarthrosis *n* = 12; hemarthrosis *n* = 8) and ACL + traumatic meniscal tear (ACL + M: hydrarthrosis *n* = 12; hemarthrosis *n* = 8) groups. Comparisons between groups were conducted using ordinal logistic regression model. Data are plotted as individual points (pg/mL), with the median and interquartile range (IQR, q1–q3) highlighted on the graph  ^∗^ *p* < 0.05.

Synovial fluid appearance was associated with differences in biomarker concentrations (Tables 3 and 4; Figure 2). Compared with hydrarthrosis, hemarthrosis was associated with higher PK2 and IL‐10 levels, whereas no statistically significant difference was observed for TNF‐α. In the ordinal logistic regression model, the odds of exceeding any given biomarker threshold were significantly different between hemarthrosis and hydrarthrosis for PK2 (OR 0.21, 95% CI: 0.06–0.75; *p* = 0.016) and IL‐10 (OR 0.09, 95% CI: 0.04–0.35; *p* < 0.0001), both remaining statistically significant after Bonferroni correction using a corrected threshold of 0.05/3. No statistically significant association was observed for TNF‐α (OR 1.28, 95% CI: 0.42–3.89; *p* = 0.665).

### 3.2. Effect of Time Postinjury on PK2 and Cytokine Levels

When biomarker concentrations were analyzed according to time from injury, descriptive data suggested a progressive decrease in PK2 and IL‐10 levels and a possible increase in TNF‐α levels from the acute to the chronic phase (Tables 3 and 4). However, ordinal logistic regression showed that time from injury, analyzed as a continuous variable, was not significantly associated with PK2 levels (OR 0.99, 95% CI: 0.98–1.01; *p* = 0.385) or TNF‐α levels (OR 1.003, 95% CI: 0.99–1.02; *p* = 0.703). In contrast, longer time from injury was significantly associated with lower IL‐10 levels (OR 0.97, 95% CI: 0.96–0.99; *p* = 0.002), and this association remained statistically significant after Bonferroni correction using a corrected significance threshold of 0.05/3. The direction of the odds ratio indicates a reduction in IL‐10 levels with increasing time from injury.

The time from injury was longer in the ACL + M group than in the isolated ACL group and, within both groups, was longer in patients with hydrarthrosis than in those with hemarthrosis (Table 5 and Figure 2). Ordinal logistic regression confirmed that increasing time from injury was significantly associated with a higher probability of hydrarthrosis. For each additional day after injury, the odds of presenting with hydrarthrosis increased by 6% (OR 1.06, 95% CI: 1.02–1.10; *p* = 0.0007). Group membership and interaction between group membership and time postinjury did not show a significant effect.

**Table 5:** Time postinjury continuous variable presented as mean, median, standard deviation, and quartiles.

	Time postinjury (days)																	
	ACL group (*n* = 20)						ACL+M group (*n* = 20)						Total (*n* = 40)					
	*n*	Mean	Median	sd	q1	q3	*n*	Mean	Median	sd	q1	q3	*n*	Mean	Median	sd	q1	q3
Time postinjury (days)	19	61.0	32	94.6	1	64	20	76.7	49	102.6	35	68	39	69.0	41	97.8	30	66
Hydrarthrosis	11	99.2	62	110.4	34	132	12	99.7	63	129.1	38	101	23	99.4	62	117.8	35	109
Hemarthrosis	8	8.5	1	13.7	0	18	8	42.1	38	11.0	34	52	16	25.3	31	21.1	1	38

Because time from injury differed according to injury type and synovial fluid appearance, group membership and synovial fluid appearance were included in the ordinal logistic regression model evaluating IL‐10 levels as potential confounding factors. After adjustment, the association between time from injury and IL‐10 levels was attenuated and showed only a borderline residual effect (OR 0.98, 95% CI: 0.97–1.00; *p* = 0.068). ACL + M status was not significantly associated with IL‐10 levels (OR 0.87, 95% CI: 0.27–2.79; *p* = 0.812), whereas hemarthrosis versus hydrarthrosis remained significantly associated with IL‐10 levels (OR = 0.20, 95% CI: 0.05;0.86, *p* = 0.031).

### 3.3. Correlation of PK2 Levels With IL‐10 and TNF‐α

**Figure 3:**
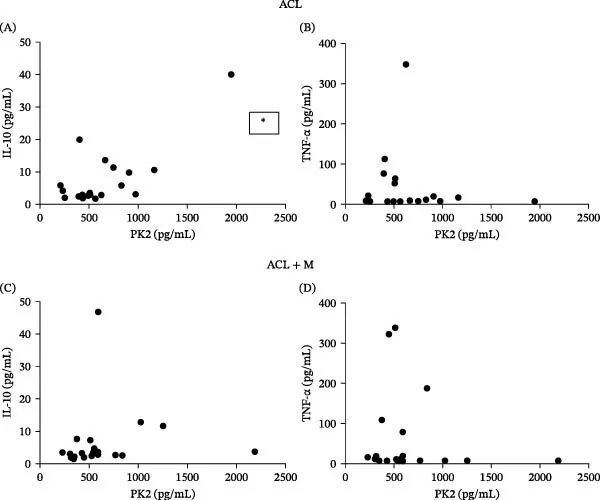
Correlation analysis of cytokines and PK2 in synovial fluid. (A) and (C) depict the correlation between PK2 (pg/mL) and IL‐10 (pg/mL) levels in ACL (*n* = 20) and ACL + M groups (*n* = 20), respectively. (B) and (D) show the correlation between PK2 (pg/mL) and TNF‐alpha (*α*) (pg/mL) levels in ACL and ACL + M groups, respectively. Correlation coefficient *r*, confidential interval CI, and *p*‐value are: A: *r*: 0.749 95% CI: 0.458–0.895 *p* = 0.0001.  ^∗^ *p* < 0.05.

To investigate potential associations between these molecules, correlation analyses were performed for both patient groups. A significant positive correlation was observed between PK2 and IL‐10 levels in ACL group (*p* < 0.05, *r* = 0.749, Figure 3A), whereas no correlation was found in ACL + M group (Figure 3C). No correlation was found between TNF‐α and PK2 levels in either group (Figure 3B,D).

### 3.4. Correlations Between Cytokine Levels and PROMs

**Figure 4:**
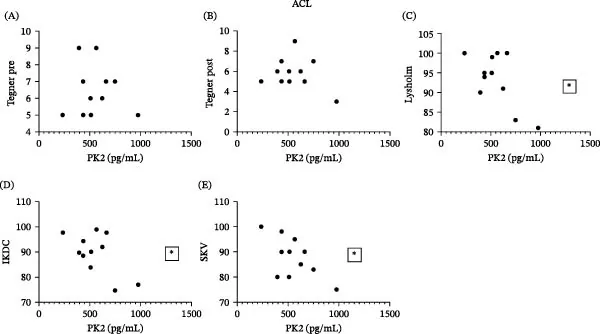
Correlation of PK2 and PROMs. Panels depict the correlation between PK2 (pg/mL) and PROMs in ACL group (*n* = 11). Tegner activity scale pre‐ (A) and postinjury (B), Lysholm knee score (C), IKDC 2000 subjective knee evaluation form (D), and simple knee value (E). Correlation coefficient *r*, confidential interval CI, and *p*‐value are: (C): *r*: −0.663 95% CI: −0.903 to −0.104 *p* : 0.026; (D): *r*: −0.613 95% CI: −0.887 to −0.021 *p* : 0.045; (E): *r*: −0.637 95% CI: −0.895 to −0.061 *p* : 0.035.  ^∗^ *p* < 0.05.

To assess potential associations between initial cytokine levels and long‐term outcomes, patients underwent follow‐up evaluations approximately 5 years post‐initial visit. A significant negative correlation was found between baseline PK2 and Lysholm, IKDC, and SKV in the ACL group (Figure 4C–E): high initial PK2 levels were associated with lower PROMs scores at follow‐up, suggesting a possible association between higher early PK2 levels and poorer patient‐reported outcomes. No correlation was found between the patients’ pre‐ and postoperative sports activity levels, as assessed by the Tegner Activity Scale (Figure 4A,B). In contrast, no association was found between PK2 levels and PROMs scores in the ACL + M group (data not shown).

## 4. Discussion

PK2 has been identified in the synovial fluid of patients with knee pathologies, including OA [24] and traumatic meniscal injuries [14], but no prior studies have explored its presence and role in acute ligamentous injuries. This study presents the first evidence that PK2 is present in the synovial fluid of patients with isolated ACL tears and combined ACL‐meniscal injuries. From previous studies, it was demonstrated that PK2 can be produced by both synoviocytes and immune cells within the joint and is usually upregulated in the presence of injury [15, 25, 26].

An increase of PK2 in the rodent knee articular joint was described in different experimental models of OA, and this overexpression was correlated to arthritis severity [25, 26]. Our group recently described an increase of PK2 in the knee joint of OA mice in the presence of an upregulation of several inflammatory markers like cytokines and metalloprotease involved in cartilage degeneration [15]. PK2 may contribute to a self‐perpetuating inflammatory cycle by attracting additional inflammatory cells that express PK2 receptors [27].

Initially, we hypothesized that patients with combined ACL and meniscal injuries (ACL+M group) would exhibit higher PK2 levels compared to those with isolated ACL tears (ACL group) due to the increased tissue damage [28]. However, our findings did not support this hypothesis. Both groups displayed similar PK2 levels, suggesting that shared inflammatory pathways may be involved in both types of injury. While these results suggest a potential role for PK2 in the inflammatory response to both types of injuries, further research is needed to fully elucidate its precise contribution to the pathophysiology of this condition.

Our analysis of TNF‐α and IL‐10, two pivotal pro‐ and anti‐inflammatory cytokines, revealed no significant differences between the two groups. This contrasts with Brophy’s findings, which reported increased TNF‐α expression in association with meniscal injuries [29]. However, the variability in cytokine measurements and the small sample size in their study may account for this discrepancy. A larger sample size could potentially clarify this difference. Nevertheless, the elevated expression of inflammatory markers highlights the increased inflammatory response associated with combined ACL and meniscal injuries, which may contribute to the accelerated breakdown of the extracellular matrix.

Our analysis revealed that demographic factors such as age, sex, weight, and height did not significantly influence synovial fluid PK2 levels. While these factors are often associated with OA risk, they do not appear to impact the inflammatory response following acute knee injury. This suggests that the inflammatory environment following joint trauma may be primarily driven by the injury itself rather than individual patient characteristics.

Our study found higher PK2 levels in ACL patients with hemarthrosis compared to those with hydrarthrosis. Hemarthrosis, characterized by increased blood and inflammatory cell infiltration, may contribute to elevated PK2 levels. Recent studies have linked elevated PK2 levels in peripheral blood to inflammatory conditions, suggesting a systemic role for this cytokine [30]. Hemarthrosis is predominantly associated with ACL injuries, yet only 12% of cases involve an isolated ACL tear, with meniscal injuries being the most common associated lesion [31]. We previously demonstrated that in patients with meniscal injuries, higher PK2 levels are also associated with hemarthrosis compared to serous effusion tears [14]. These findings highlight the complex interplay between injury type, inflammatory mediator expression, and potential pathophysiological differences between isolated and combined injuries. Interestingly, IL‐10 levels were also elevated in the hemarthrosis subgroup. This suggests a potential upregulation of anti‐inflammatory mechanisms in response to increased intra‐articular blood flow. However, blood contamination may introduce matrix effects in synovial fluid biomarker measurements and may partly reflect injury severity rather than a biomarker‐specific inflammatory pathway. Therefore, findings related to hemarthrosis should be interpreted with caution.

Our study revealed a positive correlation between PK2 and IL‐10 levels in patients with isolated ACL injuries. IL‐10 is an anti‐inflammatory cytokine that has been shown to counteract proinflammatory mediators and protect cartilage. Elevated IL‐10 levels may indicate a robust anti‐inflammatory response, potentially mitigating tissue damage. Previous research by Turati et al. [14] has also reported a correlation between PK2 and IL‐10 in patients with traumatic knee injuries, particularly those with isolated meniscal tears. However, this correlation was not observed in our cohort of patients with combined ACL and meniscal injuries. This suggests a potentially distinct inflammatory profile in cases involving multiple structural injuries. While PK2 has been implicated in both pro‐ and anti‐inflammatory processes [24], our findings highlight the complex interplay between inflammatory mediators and the potential for varying responses depending on the specific injury pattern. Further research is needed to elucidate these mechanisms and their implications for post‐traumatic joint health.

In our exploratory analysis, time from injury was not significantly associated with PK2 levels, suggesting that PK2 concentrations may not follow a clear time‐dependent pattern in this cohort. This observation may be consistent with a sustained inflammatory process and ongoing PK2 production in the synovial fluid, possibly due to the activation of different cell types. Previous research on meniscal tears and OA has also shown persistent PK2 levels, supporting the hypothesis of a prolonged inflammatory role for this cytokine [14]: PK2 may play a continuous role in the inflammatory environment of joint disorders rather than exhibiting a transient or time‐dependent expression pattern. In contrast, we observed a decreasing kinetic pattern for IL‐10, with higher levels in the acute phase [16]. This anti‐inflammatory cytokine showed higher levels shortly after the injury, aligning with its well‐documented role in promoting early tissue repair and counteracting proinflammatory mediators. However, IL‐10 levels declined at a later time, indicating a diminished anti‐inflammatory response in the chronic phase. These observations reinforce the hypothesis that IL‐10 is activated in the acute phase to mitigate damage and promote repair, while its downregulation in later stages might contribute to an imbalance favoring a prolonged inflammatory state. These findings highlight the complex interplay between proinflammatory and anti‐inflammatory cytokines in the context of knee injuries. Further investigation is needed to elucidate the precise mechanisms underlying these temporal dynamics and their implications for long‐term joint health.

At the 5‐year follow‐up, patients completed four PROMs. The negative correlation between PK2 levels and IKDC, Lysholm, and SKV scores suggests a possible association between higher early PK2 concentrations and poorer long‐term clinical outcomes. This is consistent with the hypothesis that elevated PK2 levels may be associated with joint dysfunction through its proinflammatory effects and influence on pain perception. The role of PK2 in nociceptive sensitization, a key factor in acute and chronic knee conditions, is well established. By directly activating nociceptors or promoting the release of pain‐inducing mediators like CGRP, substance P, and cytokines, PK2 can enhance pain sensitivity [27, 32]. This heightened inflammatory response and increased pain perception may lead to impaired functional recovery, reduced patient satisfaction, and delayed return to sports, as reflected by lower PROM scores. Consistent with this, several animal models of OA have shown that the inhibition of the PK2 system with pharmacological antagonists can reduce local inflammation and tissue damage, restoring motor function and preventing pain development [15, 25, 26].

While this study provides valuable insights into the potential role of PK2 in the post‐traumatic inflammatory environment following ACL injury, some limitations should be acknowledged. The relatively small sample size, the single‐center design, the inherent variability of cytokine measurements, and the difficulty of obtaining synovial fluid from healthy controls limit the generalizability of our findings. Moreover, follow‐up was based on clinical outcomes only, without a radiographic or MRI‐based assessment of structural PTOA. Another limitation is the heterogeneity in sampling context, as synovial fluid was collected either at initial presentation or intraoperatively during arthroscopy: although all samples were collected and processed at the same time using the same standardized protocol and time from injury was analyzed as a continuous variable, the potential influence of sampling context cannot be fully excluded. Furthermore, residual confounding cannot be excluded as the limited sample size did not allow robust adjustment for all potentially relevant clinical variables, including rehabilitation protocol, time to surgery, baseline activity level, and meniscal treatment characteristics.

Future longitudinal studies with larger sample sizes, radiographic or MRI‐based structural assessment, and mechanistic analyses are needed to further explore the temporal dynamics of PK2 expression and its association with clinical outcomes and PTOA‐related structural changes.

## 5. Conclusion

This study investigated the presence of PK2, a chemokine‐like peptide involved in inflammatory and pain‐related processes, in synovial fluid following ACL injury.

Although we recognize the exploratory nature of this study and the need for additional confirmatory analyses, our findings provide novel evidence supporting the potential contribution of PK2 in the inflammatory response associated with ACL trauma. PK2 was detectable in the synovial fluid across different phases of injury, suggesting its possible involvement in the inflammatory response after ACL injury.

Further longitudinal studies including larger cohorts, radiographic or MRI‐based assessment of PTOA, and mechanistic analyses are needed to clarify the biological and clinical relevance of PK2 after ACL injury.

## Author Contributions

**Marco Crippa**: conceptualization, investigation, methodology, writing – original draft. **Silvia Franchi and Paola Sacerdote**: conceptualization, formal analysis, investigation, visualization, writing – review and editing. **Laura Rizzi**: conceptualization, investigation, visualization, writing – original draft. **Giulia Galimberti and Giada Amodeo**: formal analysis, investigation, visualization. **Laura Antolini**: formal analysis, investigation, methodology, visualization, writing – review and editing, supervision. **Massimiliano Piatti**: investigation, resources. **Nicola Portinaro**: conceptualization, methodology. **Giovanni Zatti:** funding acquisition, investigation, methodology, project administration, resources, supervision. **Antonio Torsello**: conceptualization, funding acquisition, investigation, methodology, project administration, resources, supervision. **Marco Turati**: conceptualization, investigation, methodology, project administration, resources, writing – review and editing. **Marco Bigoni**: conceptualization, investigation, methodology, project administration, resources, supervision.

## Funding

This study was supported in part by the Fondo di Ateneo per la Ricerca of the University of Milano‐Bicocca. Open access publishing facilitated by Universita degli Studi di Milano‐Bicocca, as part of the Wiley—CRUI‐CARE agreement.

## Ethics Statement

The study was conducted in accordance with the Declaration of Helsinki and approved by the Ethics Committee of the Fondazione IRCCS San Gerardo dei Tintori.

## Consent

Informed consent was obtained from all subjects involved in the study.

## Conflicts of Interest

The authors declare no conflicts of interest.

## Data Availability

The data that support the findings of this study are available upon request from the corresponding author. The data are not publicly available due to privacy or ethical restrictions.
